# Farming practices in Sweden related to feeding milk and colostrum from cows treated with antimicrobials to dairy calves

**DOI:** 10.1186/1751-0147-55-49

**Published:** 2013-07-09

**Authors:** Anna Duse, Karin Persson Waller, Ulf Emanuelson, Helle Ericsson Unnerstad, Ylva Persson, Björn Bengtsson

**Affiliations:** 1 Department of Animal Health and Antimicrobial Strategies, National Veterinary Institute, Ulls väg 2B, Uppsala SE-751 89, Sweden; 2 Department of Clinical Sciences, Swedish University of Agricultural Sciences, Ulls väg 14C, Uppsala SE-756 51, Sweden; 3 Växa Sverige, Box 288, Uppsala, SE-75105, Sweden

**Keywords:** Calves, Antibiotics, Milk withdrawal, Dry cow treatment, Feeding, Non-saleable milk, Mastitic milk

## Abstract

**Background:**

Milk produced by cows in receipt of antimicrobial therapy may contain antimicrobial residues. Such antimicrobial-containing waste milk must be withdrawn from human consumption and is therefore sometimes used as calf feed. Unfortunately, this approach might promote selection of antimicrobial resistant bacteria in the calves’ intestinal microbiota. The objectives of this study were therefore to obtain an overview of waste milk feeding practices on Swedish dairy farms and to investigate if these practices were associated with certain farm characteristics. A representative group of 457 Swedish dairy farmers participated in a web-based survey with questions about the use of colostrum and milk from cows treated with antimicrobials at dry off or during lactation, respectively, as calf feed.

**Results:**

Colostrum (milk from the first milking after calving) and transition milk (milk from the second milking to the fourth day after calving) from cows treated with antimicrobials at dry off was fed to calves on 89% and 85% of the farms in the study, respectively. When antimicrobial therapy was given to cows during lactation, 56% of the farms fed milk that was produced during the course of treatment to calves, whereas milk that was produced during the subsequent withdrawal period was fed to calves on 79% of the farms. Surveyed farmers were less prone to feed such milk if the antimicrobial therapy was due to mastitis than other infections. In Sweden, a majority of antimicrobial treatments during lactation are systemic administration of benzylpenicillin and thus, the bulk of waste milk in Sweden is likely to contain residues of this drug. Feeding waste milk to calves was more common on non-organic farms, and on farms located in Southern Sweden, and was less common on farms with cows housed in cold free stalls barns.

**Conclusions:**

Waste milk that may contain antimicrobial residues is, at least occasionally, used as feed for calves on a majority of surveyed Swedish dairy farms. Future work should focus on the effect of waste milk feeding on the occurrence of antimicrobial resistant bacteria in the calves’ intestinal microbiota.

## Background

Milk produced by cows in receipt of antimicrobial therapy may contain antimicrobial residues. Such antimicrobial-containing waste milk must be withdrawn from human consumption during the course of treatment as well as during the statutory withdrawal period. The withdrawal period is set by the national Medical Products Agency or the European Medicines Agency, and is defined as the time necessary for the residues of an administered product to decrease below the Maximum Residue Limit (MRL) [1,2]. Discarding waste milk inevitably leads to reduced income and logistical problems with the disposal of large quantities of milk. To mitigate some of these losses and to avoid the disposal issues, farmers sometimes use waste milk as feed for calves.

The use of waste milk as calf feed is controversial. Concerns have mostly been raised in regard to a decreased hygienic quality or the role of waste milk as a vector for different pathogens [3,4]. As the emergence of antimicrobial resistance has accelerated in recent years [5], more focus has been directed towards the content of antimicrobial residues in waste milk. It has been suggested that such residues may impose a selection pressure on the calf’s intestinal microbiota if waste milk is ingested by the calf. This may increase the prevalence of resistant bacteria in the calf’s intestines. A few studies have touched upon this issue but the results are inconclusive. In a study by Wray *et al.*[6], fecal *Escherichia coli* (*E. coli)* was monitored for antibiotic resistance. In the first trial, a significantly higher Minimum Inhibitory Concentration (MIC) of streptomycin was found for *E. coli* from calves fed antimicrobial-containing milk, compared to calves fed milk replacer, but a significant difference in MIC was not observed for ampicillin [6]. In the second trial, no difference was observed between the susceptibility of *E. coli* from calves fed antimicrobial-containing milk and control calves [6]. In another study, a higher prevalence of resistant *E. coli* was found in calves fed non-pasteurized waste milk compared to calves fed bulk tank milk, but no differences were found in the proportion of resistant *Enterococci*[7]. Thus, there is a need for further research before definite conclusions can be drawn about the effect of waste milk feeding on the intestinal microbiota of the calves.

Despite the concerns above, there are no regulations on waste milk feeding on non-organic farms. In the European Union, the disposal of animal waste is regulated by the Animal-By-Products regulation (EC) No 1069/2009, but milk or colostrum that is used and disposed on the farm of origin is not covered by this regulation [8]. However, organic farmers in Sweden have to apply a withdrawal period extended beyond the statutory withdrawal period. On these farms, milk or colostrum from cows in receipt of antimicrobials must only be fed to calves during the extended withdrawal period, with the exception of colostrum and transition milk to the dam’s own newborn calf [9]. Without any formal regulations, it is assumed that the choice of feeding waste milk to calves is predominantly based upon each farmer’s experiences and traditions.

Until now, routines for waste milk feeding have not been surveyed on Swedish dairy farms. The primary objective of this study was therefore to obtain an overview of management practices related to feeding of colostrum and milk from cows treated with antimicrobials to calves on Swedish dairy farms. A second objective was to investigate if these management practices were associated with certain farm characteristics such as non-organic versus organic farming, geographic location, housing system and herd size.

## Methods

### Dairy farms

A list of Swedish dairy farms with a registered e-mail address was provided by the Swedish Dairy Association. The list contained e-mail addresses to 1735 unique farms (33% of Swedish dairy farms in 2011 [10]). Even though registration of e-mail addresses is voluntary for the farmer, the group of farmers with an email address was a representative sample of Swedish dairy farms in regards to geographic location and herd size [10,11].

### Questionnaire development

A questionnaire was developed and implemented electronically using the online survey platform Easyresearch (QuestBack™) [12]. All information that was obtained via the platform was owned by the authors of this paper, and measures were taken to ensure that the e-mail addresses of the farmers were protected. The questionnaire consisted mainly of multiple-choice and semi-closed questions as well as a few matrix questions. It was developed with conditional branching to follow-up questions that was based on the answer to the previous question. The questionnaire was divided into three parts; basic farm data (herd ID, herd size and non-organic versus organic farming), management of colostrum and transition milk from cows treated with antimicrobials at dry off and management of milk from cows treated with antimicrobials during lactation. In Sweden, the use of antimicrobials for animals is strictly regulated and all antimicrobials for use in animals are only available from the veterinarian directly, or on veterinary prescription [13]. A majority of treatments of dairy cows in Sweden are systemic treatments with benzylpenicillin or dry cow treatment with benzylpenicillin in combination with aminoglycosides [14]. Thus, in order to simplify the questionnaire, questions in the survey did not differentiate between waste milk feeding routines after treatments with different antimicrobial compounds and different administration routes.

The study was conducted solely as a questionnaire survey to farmers and did not include experiments on animals. The study could therefore be conducted without ethical approval for animal experiments. The questionnaire did not contain any questions that could interfere with the personal integrity and thus, there was no need for an ethical approval for research on humans. Further, every precaution was taken to keep the personal information of the farmers confidential and to minimize the impact on their integrity according to the Helsinki Declaration.

A link to the survey and an accompanying electronic letter that contained information about the purpose of the study and assured confidentiality to participants was sent by email on May 2^nd^ 2011 to 1735 farms. An URL link to the questionnaire was also provided on the official homepage of the Swedish Dairy Association, which gave other farmers that were not on the list an opportunity to take part in the survey. Automatic reminders were sent out to non-respondents once every week for four weeks. The survey was closed on June 6^th^ 2011. As an incentive for completion, all respondents were promised to receive a summary of the results when the survey was completed. No other incentives were given. The questionnaire is available, in Swedish, upon request from the first author.

### Data analyses

Results from the questionnaire were automatically exported from the questionnaire platform to a Microsoft Excel 2010 spreadsheet (Microsoft Corporation, Seattle, WA, USA). Additional data on geographic location as well as on housing system for each herd was collected from the Swedish Official Milk Recording Scheme. For the purpose of this paper, waste colostrum (WC) is defined as colostrum from the first milking after calving, produced by a cow that was treated with antimicrobials at dry off. Waste transition milk (WT) is defined as milk from the second milking until the fourth day post partum that is produced by a cow treated with antimicrobials at dry off. When a cow is treated with antimicrobials during lactation, the milk must be withdrawn during both the course of treatment and the following withdrawal period. Milk that is produced during the course of treatment is here defined as treatment waste milk (TWM). Milk that is produced from the first day after treatment has stopped until the day the milk can be sold for human consumption again is defined as withdrawal waste milk (WWM).

Descriptive statistics were presented as the absolute number of farms, as well as the proportion of farms in the survey, giving a particular response. Additionally, the most important comments to semi-closed questions were presented, if feasible. The dairy herds were categorized based on their size (<60 cows, between 60 and 89 cows, and ≥90 cows), with thresholds chosen in order to obtain even groups. The farms geographic location was defined by the highest subdivision (lands/NUTS1) using the Nomenclature of Territorial Units for Statistics [15] (Eastern Sweden, Southern Sweden including the islands, and Northern Sweden). Finally, the farms were also categorized according to housing system (warm free stall, cold free stall, and tie stall). The Wilcoxon’s rank sum test and Fisher’s exact test were used to assess the similarity of respondents to non-respondents regarding herd size and geographic location. Furthermore, the associations between basic farm data (herd size, production type, housing type, and geographic location) and survey response variables were analysed using Fisher’s exact test. All statistics were conducted in Stata 11 (StataCorp LP, 2011; Stata Statistical Software: Release 11.2; College Station, TX, USA: StataCorp LP). Associations according to p < 0.05 were considered significant throughout the analyses.

## Results

### Characteristics of respondents and non-respondents

One hundred and sixty-eight (10%) of the total 1735 farms that were e-mailed could not be contacted via the provided e-mail addresses. Of the remaining 1567 farms, 479 (31%) completed the questionnaire. Forty-three (3%) of the contacted farms reported that they could not participate in the survey because they no longer kept a dairy herd and the remaining 1045 (67%) farms did not respond at all. Thirty-seven of the 479 responding farms completed less than 60% of the questionnaire and were thus excluded from the survey. Finally, fifteen additional farmers took the opportunity to participate in the survey via the URL-link on the official homepage of the Swedish Dairy Association. In total, answers from 457 dairy farmers were included in the study.

Of the 455 farms that reported herd size in the questionnaire, defined as the total number of lactating and dry cows, herd size ranged from 5 to 1030 cows and the mean was 96 cows (95% CI: 88 to 104). Median herd size was 68 cows for respondents and 67 cows for non-respondents. Twenty per cent, 54% and 26% of responding farms were located in Eastern Sweden, Southern Sweden and the islands and Northern Sweden, respectively, whereas for non-responding farms these numbers were 17, 55 and 28%, respectively (data available for 441 farms). Respondents and non-respondents were not significantly different regarding herd size (p = 0.546) and geographic location (p = 0.455). Eighty-one per cent of the surveyed farmers were non-organic farmers, 18% were organic farmers certified by KRAV (an organisation for organic farming in Sweden) [9] and 1% were certified by another organisation for organic farming (data available for 454 farms). On 41% of the farms, cows were housed in warm free stall barns, while 11% housed cows in cold free stall barns and the remaining 48% kept their cows in a tie stall barn (data available for 412 farms).

### Feeding calves waste colostrum and waste transition milk

WC was, at least occasionally, fed to calves on 89% of the farms (Table 1). If WC was not always used as calf feed, the most common approach was to only feed WC if it was of sufficient quality or if no alternative colostrum was available. WT was, at least occasionally, fed to calves on 85% of the farms (Table 2). If WT was not always used as calf feed, the most common approach was to feed it only to certain calves, in particular to bull calves.

**Table 1:** Feeding calves colostrum from cows treated with antimicrobials at dry off

**Are calves fed colostrum (WC) from cows treated with antimicrobials at dry off?**	**Number of farms**	**Proportion of farms**^**1 **^**(%)**
Always	207	45
Only to heifer calves	0	0
Only to bull calves	18	4
Only if alternative colostrum is not available	84	19
Only if it is of sufficient quality	100	22
Never	49	11
Not responding	1	0
Other reason^2^	19	4

^1^ Proportions add to more than 100% as some farmers ticked multiple answers.

^2^ Some farmers stated that they never or rarely use antimicrobials at dry off, that colostrum from the treated dam is only given to her own newborn calf, that calves are given such colostrum only if they were accidently left with the dam or that they are obliged to let the newborn calf suckle its dam according to the rules for organic certification.

**Table 2:** **Feeding calves transition milk**^**1 **^**from cows treated with antimicrobials at dry off**

**Are calves fed transition waste milk (WT) from cows treated with antimicrobials at dry off?**	**Number of farms**	**Proportion of farms**^**2 **^**(%)**
Always	260	58
Only to certain calves^3^	84	19
*Heifer calves*	*10*	*12*
*Bull calves*	*64*	*76*
*Calves above a certain age*^*a*^	*10*	*12*
*Calves below a certain age*^*b*^	*6*	*7*
*Other*^*4*^	*14*	*17*
*No answer*	*0*	*0*
Only from a particular day after calving	42	9
*The day after calving*	*2*	*5*
*Two days after calving*	*5*	*12*
*Three days after calving*	*17*	*43*
*Four days after calving*	*16*	*40*
Never	66	15
Not responding	3	1

^1^ Transition milk is milk from the second milk to the fourth day after calving.

^2^ Proportions add to more than 100% as some farmers ticked multiple answers.

^3^ This question was followed by a follow-up question only seen by respondents choosing this option. The options and responses for the follow-up question are shown in italic.

^a^ Median = 4 weeks, mean = 4.5 weeks, ^b^ Median = 1 week, mean = 1.2 weeks.

^4^ Some responded that TWM is only fed to the dam’s own calf because the calf and cow are kept together during this period. Others stated that they are only allowed to feed TWM to the dam’s own calf according to the rules for organic certification or that fresh cows are used as nursing cows for a group of calves during this period.

### Feeding calves waste milk from cows treated with antimicrobials during lactation

Treatment waste milk (TWM) was, at least occasionally, fed to calves on 56% of the farms (Table 3). If TWM was not always used as calf feed, most farmers reported that it was fed to calves only if it was produced by cows treated for certain diseases. Farmers were less prone to feed TWM from cows treated for mastitis than from cows treated for other infections. Withdrawal waste milk (WWM) was, at least occasionally, fed to calves on 79% of the farms. If WWM was not always used as calf feed, it was most often fed only to certain calves, predominantly bull calves. Only thirty farms (7%) reported that treated cows are used, at least occasionally, as nursing cows. Some of farmers reported that treated cows are used as nursing cows only if the cow was a nursing cow before she was treated or if she was destined to be a nursing cow after the withdrawal period (data not shown).

**Table 3:** Feeding calves milk produced by cows given antimicrobials during lactation, during treatment and withdrawal periods

**Are calves fed waste milk from cows treated with antimicrobials during lactation?**	** Treatment period (TWM)**		** Withdrawal period (WWM)**	
	**Number of farms**	**Proportion of farms**^**1 **^**(%)**	**Number of farms**	**Proportion of farms**^**1 **^**(%)**
Always	92	21	184	41
Only to certain calves^2^	68	15	81	18
*Heifer calves*	*5*	*8*	*9*	*12*
*Bull calves*	*62*	*94*	*74*	*95*
*Calves above a certain age*	*11*^*a*^	*17*	*6*^*b*^	*8*
*Calves below a certain age*	*3*^*c*^	*5*	*5*^*c*^	*6*
*Other*^*4*^	*4*	*6*	*3*	*3*
*No answer*	*2*	*0*	*3*	*0*
Only from cows treated for certain diseases^2,3^	74	17	55	12
*Mastitis*	*31*	*42*	*25*	*47*
*Uterine infection*	*51*	*70*	*44*	*83*
*Infections in legs and hooves*	*69*	*95*	*48*	*91*
*Other diseases*	*43*	*59*	*37*	*70*
*No answer*	*1*	*0*	*2*	*0*
Only during part of the period^4^	55	12	63	14
Never	199	44	93	21
Not responding	9	2	12	3

^1^ Proportions add to more than 100% as some farmers ticked multiple answers.

^2^ This question was followed by a follow-up question only seen by respondents choosing this option. The options and responses for the follow-up question are shown in italic.

^a^ Median = 4 weeks, mean = 4.8 weeks ^b^ Median = 4 weeks, mean = 3.7 weeks ^c^ Median = 2 week, mean = 2 weeks.

^3^ Many farmers stated that they feed calves TWM only if it resembles normal milk, others that such milk is discarded if the cow is infected with certain bacteria, mostly *Escherichia coli* or *Staphylococcus aureus*, or if the cow is treated with a certain type of antimicrobial.

^4^ Some of the organic farmers reported that they only feed calves waste milk produced after the statutory withdrawal period, according to the rules for organic certification.

### Farm characteristics associated with waste milk feeding

As seen in Table 4, WC, WT, TWM or WWM were all more often fed to calves on non-organic farms than on organic farms (p = 0.019, p < 0.001, p < 0.001 and p < 0.001, respectively). WC was fed to calves on significantly more warm free stall farms (p = 0.011) or tie stall farms (p = 0.007) compared to cold free stall farms. WT, TWM or WWM were all more often fed to calves on tie stall farms compared to cold free stall farms (p = 0.031, p = 0.031 and p = 0.006, respectively). Feeding TWM or WWM was significantly more common on farms located in Southern Sweden and on the islands compared to farms in Northern Sweden (p < 0.001) or Eastern Sweden (TWM: p < 0.001 and WWM: p = 0.001). However, feeding WC or WT was independent of geographical region and the proportion of farms feeding WC, WT, TWM or WWM was not associated with different herd sizes.

**Table 4:** Proportions of farms with different farm characteristics that feed calves different types of waste milk

**Farm characteristics**	**Waste milk feeding**							
	**WC**^**1**^		**WT**^**2**^		**TWM**^**3**^		**WWM**^**4**^	
	**% (n**^**5**^**)**	**p-value**^**6**^	**% (n)**	**p-value**	**% (n)**	**p-value**	**% (n)**	**p-value**
Production type:								
Organic	81 (86)	Ref.	67 (86)	Ref.	26 (84)	Ref.	58 (84)	Ref.
Non-organic	91(365)	0.019	90 (365)	<0.001	62 (361)	<0.001	84 (358)	<0.001
Herd size:								
≥90 cows	90 (154)	Ref.	84 (157)	Ref.	52 (155)	Ref.	76 (154)	Ref.
60 – 89 cows	87 (150)	0.469	84 (148)	1.000	55 (146)	0.644	80 (145)	0.407
<60 cows	90 (148)	1.000	88 (147)	0.319	59 (145)	0.245	81 (144)	0.323
Housing:								
Cold free stall	76 (46)	Ref.	76 (46)	Ref.	42 (45)	Ref.	64 (44)	Ref.
Warm free stall	91 (165)	0.011	84 (167)	0.276	54 (167)	0.182	78 (165)	0.052
Tie stall	92 (198)	0.007	89 (197)	0.031	60 (193)	0.031	84 (194)	0.006
Geographic location:								
Southern Sweden and the Islands	89 (236)	Ref.	86 (237)	Ref.	67 (233)	Ref.	87 (234)	Ref.
Northern Sweden	88 (116)	0.719	88 (116)	0.739	43 (115)	<0.001	70 (115)	<0.001
Eastern Sweden	93 (86)	0.398	81 (85)	0.294	44 (84)	<0.001	69 (81)	0.001

^1^ Colostrum from the first milking after calving, produced by cows treated with antimicrobials at dry off.

^2^ Transition milk from cows treated with antimicrobials at dry off, produced from the second milking to the fourth day after calving.

^3^ Milk produced during the course of treatment by a cow that was given antimicrobial therapy during lactation.

^4^ Milk produced during the withdrawal period by a cow that was given antimicrobial therapy during lactation.

^5^ Number of farms in each category for which data on waste milk feeding and farm characteristics is available.

^6^ A p-value < 0.05 indicates significant differences between the reference level and other levels within each farm characteristic (Fisher’s exact test).

## Discussion

This is the first survey in Sweden reporting on the use of waste milk from cows treated with antimicrobials as feed for calves. It was revealed that dairy calves, at least occasionally, are fed waste milk on a majority of the farms, but the use of waste milk as calf feed varies for different types of farms.

### Feeding calves waste colostrum and waste transition milk

Calves are, at least occasionally exposed to WC on 89% of the farms, which is similar to surveyed farms in the United Kingdom (UK), where 93% of farms that feed waste milk reported that such milk may contain colostrum from cows treated with antimicrobials at dry off [16]. Only about 30% of the Swedish dairy cows are treated with antimicrobials at dry off [14], in comparison to the UK farms, where nearly 75% reported that their first choice dry cow antimicrobial tube is given to 81-100% of the cows in the herd [16]. Another difference between these countries is the availability of products for dry cow therapy. In Sweden, most dry cow antimicrobials are combinations of benzylpenicillin and aminoglycosides [14], whereas in the UK, several types of dry cow antimicrobials are available [16]. Thus, not only are more calves expected to be exposed to WC in the UK than in Sweden, WC may also contain residues of a wider variety of antimicrobials.

Since all calves require colostrum at birth, storing antimicrobial-free colostrum is a prerequisite for farmers that want to avoid feeding WC. In surveys among dairy farmers in the UK, United States (US) and Canada, it was revealed that only 13, 38 and 32% of the farmers, respectively, stored frozen colostrum [16-18]. Storage of colostrum among Swedish farmers was not investigated in the survey, which is a limitation of the present study. It is likely that Swedish farmers act similarly and thus, do not store quantities of colostrum sufficient to supply all newborn calves with antimicrobial-free colostrum, although this was not further elucidated in this study. Since approximately one fifth of the farmers avoid feeding WC if alternative colostrum is available, it can be assumed that WC feeding would be less common if more farms stored colostrum from non-treated cows. Feeding WT was less common than feeding WC, which was most apparent for organic farmers. A plausible explanation for this is that organic farmers in Sweden are only allowed to feed WT from the calf’s own dam [9] and thus, for practical reason, feeding bulk milk tank might be preferred.

### Feeding calves waste milk from cows treated with antimicrobials during lactation

The proportions of farms that feed either TWM, WWM or both was comparable to the proportion of farms feeding waste milk to calves in England and Wales (83%) but much higher than what has been reported from Canada (49%) and the US (31%) [16,18,19]. Due to differences in study and questionnaire design, and that waste milk in some studies is not exclusively defined as milk from cows treated with antimicrobials, the studies might not be completely comparable. Despite this, the results indicate that fewer farms in North America compared to Sweden and the UK feed waste milk that may contain antimicrobial residues to calves. In the US, milk replacer is the main type of liquid diet fed to calves and thus, waste milk or whole milk in general seems to be fed on fewer farms than in the UK or Sweden [16,19,20]. Rather than being due to a wish to reduce the risk of exposing calves to antimicrobial residues in milk, it is more likely that this difference reflects an attempt to reduce the transmission of pathogens such as *Mycobacterium avium, subsp. paratuberculosis* (MAP) and *Mycoplasma bovis*[21-23], that are more commonly encountered on US farms than on Swedish or UK farms [24-27].

One thing that should be kept in mind is that 85% of mastitis treatments in Sweden are systemic benzylpenicillin, whereas the use of e.g. flouroquinolones and cephalosporins (ceftiofur) is less common (9.5% and 1.1%, respectively) [28]. In the UK, benzylpenicillin was used to treat mastitis on only 1% of the farms and instead, a wide variety of broader spectrum antimicrobials was commonly used [16]. Compared to most other antimicrobials, benzylpenicillin in waste milk is to some extent degraded in the gastrointestinal tract of calves and many bacterial species in the intestines of calves are intrinsically resistant to this drug [29]. Waste milk that contains benzylpenicillin is therefore less likely to affect the prevalence of resistant bacteria in the calves’ intestinal microbiota than if it contains other antimicrobials. Thus, waste milk feeding is assumed to be safer in Sweden than in the UK and in most likely other countries as well.

To the authors’ knowledge; this is the first study that separately surveys TWM and WWM feeding. In the current study, farmers were much more reluctant to feed TWM than WWM to calves. One reason for this is that fewer organic farmers in the survey used TWM than WWM as feed for calves, which is in line with the rules for organic certification [9]. Still, 26% of the organic farmers reported that TWM is used as feed for calves, despite the ban on TWM feeding on organic farms. Another plausible explanation to the difference in farms feeding TWM and WWM is that TWM is likely to contain higher residue concentrations than WWM and therefore may be less attractive as calf feed for the farmer. Although even low concentrations of antimicrobials are sufficient to cause a selection of resistant bacteria [30], the extent of compositional changes in the calves’ intestinal microbiota may be different if TWM, WWM or both is fed to calves. However, the focus of this survey was only to describe routines for waste milk feeding on Swedish dairy farms and a thorough assessment of the effect of applying these routines on the susceptibility of the calves’ intestinal microbiota was beyond the scope of this paper. This survey is part of a larger study in which the association between waste milk feeding and the occurrence of antimicrobial resistant bacteria in the calves’ intestines is investigated.

As Brunton *et al.*[16] concluded, calves might be less exposed to antimicrobial residues in waste milk if waste milk from the first milking after treatment is discarded. This is similar to the practice recommended in Sweden by Växa Sverige (former Swedish Dairy Association) (Landin 2012 – personal communication), where farmers are encouraged to discard the milk produced during the first day after treatment has stopped. However, only 36% of the Swedish farmers discard WWM always, or during part of the withdrawal period, suggesting that current recommendations in Sweden are not followed to a great extent. This result is also comparable to the 30% of the UK farms [16] that discarded the first milking after treatment.

Mastitis was a common reason to avoid feeding TWM to calves, whereas for WWM, the type of cow disease was of less importance. A possible explanation for this difference is that mastitic TWM is more likely to contain potential pathogens and to have visual changes than mastitic WWM and might therefore be considered by the farmer as inappropriate calf feed. Finally, using cows that have been given antimicrobial therapy during lactation as nursing cows for calves was only applied on 7% of the surveyed farms, and is therefore assumed to play a minor role in the disposal of TWM and WWM.

### Farm characteristics associated with waste milk feeding

In the survey, it was revealed that calves are fed waste milk on significantly fewer organic than non-organic farms. This could be expected because the rules for organic certification states that WC and WT must not be fed to other calves than the dam’s own newborn calf [9] Moreover, TWM is not permitted as calf feed and WWM can only be fed after the statutory withdrawal period [9]. A similar difference between non-organic and organic farms was not found for surveyed farms in the UK, which might be explained by the small number of organic farmers (n = 28) participating in the UK survey [16]. Feeding waste milk was also less common on farms with cold free stall barns than on farms with tie stall barns. A reason for this difference was not sought in this survey. No difference in waste milk feeding was observed when comparing farms with different numbers of cows, which was also observed among US dairy farms [19]. Farmers in the Southern parts of Sweden were more prone than farmers in other regions to feed TWM and WWM. Similar regional differences were also observed for biosecurity management practices in a survey among livestock farmers in Sweden [31]. This suggests that farmers in different regions in Sweden perceive risks differently or that similar strategies are communicated among farmers or advisory services in nearby areas.

### Response rate and questionnaire design

As for all surveys, the response rate is critical for the overall quality of the final results. The response rate in this survey can be considered low (31%), but was comparable to the response rate in a similar survey conducted in England and Wales (30%) [16], and can be considered normal for email surveys in general [32].

A limitation of the present study was that no differentiation was done between waste milk feeding routines for different antimicrobial substances or administration routes. This generalisation could, however, be justified since a majority of antimicrobial treatments of dairy cows in Sweden are systemic treatments with benzylpenicillin [14]. Therefore, it did not seem reasonable to add additional questions about specific routines for different treatment regimes. This would have increased the length of the questionnaire and the workload for the farmer, which could have adversely affected the completeness and response rate of the survey.

Survey results may be affected by self-selection and non-response bias. Self-selection bias can occur when farmers volunteer to take part in the questionnaire, whereas non-response bias occurs if respondents differ from non-respondents. Since the group of farmers that received the questionnaire was similar to the general population of Swedish dairy farmers, and non-respondents were similar to respondents, when considering geographic location and herd size, it is likely that neither self-selection nor non-response bias had a significant effect on the final results [10,11].

A clear advantage with this kind of web-based survey is the possibility to include conditional branching with follow up questions based on the answer to the previous question. Thus, respondents are not exposed to irrelevant questions, which might increase the completeness of the survey. Another advantage is that web-based surveys are less time-consuming and costly compared to mailed or telephoned questionnaires, due to for instance automatic collection and compiling of responses Thus, the questionnaire can be distributed to a large group of farmers without resulting in a heavy work load or high costs [33].

## Conclusions

This survey provides an overview of management practices related to the feeding of waste milk from cows treated with antimicrobials to calves on Swedish dairy farms. It was observed that waste milk, at least occasionally, is fed to calves on a majority of Swedish dairy farms that were surveyed. Feeding waste milk to calves was most commonly applied on non-organic farms, and on farms located in Southern Sweden, and was not so common on farms with cows housed in cold free stall barns. Future work should focus on the effect of waste milk feeding on the occurrence of antimicrobial resistant bacteria in the calves’ intestinal microbiota.

## Abbreviations

WC: Colostrum from cows treated with antimicrobials at dry off; WT: Transition milk from cows treated with antimicrobials at dry off that is produced from the second milking until the fourth day after calving; TWM: Milk from cows treated with antimicrobials during lactation and produced during the course of treatment; WWM: Milk from cows treated with antimicrobials during lactation and produced during the withdrawal period.

## Competing interests

The authors declare that they have no competing interests.

## Authors’ contributions

All authors were involved in the design of the study, the development of the questionnaire, and the review and approval of the questionnaire. AD coordinated the study, performed the data analysis and drafted the manuscript. All authors read and approved the final manuscript.
